# The Effect of Soil Moisture on the Ability to Detect TNT Pairs from the Sand Layer in Order to Prevent Environmental Pollution and Groundwater

**DOI:** 10.3390/molecules26133908

**Published:** 2021-06-26

**Authors:** Wojciech Pawłowski, Monika Karpińska

**Affiliations:** 1 Department of High-Energetic Materials, Faculty of Chemistry, Warsaw University of Technology, Noakowskiego 3, 00-664 Warsaw, Poland; wojtek@ch.pw.edu.pl; 2 The Kielanowski Institute of Animal Physiology and Nutrition, Polish Academy of Sciences, Instytucka 3, 05-110 Jabłonna, Poland

**Keywords:** environmental pollution, explosives, TNT, GC-MS, MO-2M

## Abstract

The aim of the study was to investigate the influence of sand bed moisture on TNT transport from under the sand layer. The MO-2M explosive vapor detector was used, the detection mechanism of which is based on the FAIMS method. In addition, it was determined after what time the detector alarm appears, signaling the presence of TNT vapors, and how it affects the thickness of the sand layer. The performed work allowed us to assess the suitability and possibly adapt the MO-2M detector to detect non-metal mines, which will help develop new application possibilities for this device. These tests can also be used to eliminate environmental contamination resulting from the deposition of explosives in the ground and the migration of harmful compounds to groundwater.

## 1. Introduction

Maintaining public safety is one of the main goals of world leaders today. To this day, there are many minefields and post-war remains in former battlefields, which pose a threat to the people living there. Also, the military in its current hostilities continues to put minefields as the best protection against the movement of enemy tanks, trucks and troops in the areas of military operations. Modern minefields are also meant to be dams to warn of intruders entering the guarded areas. Unfortunately, minefields pose a threat to the life and health of the local population and the environment is increasingly contaminated, as they contribute to the deposition of free explosives (including carcinogenic nitro compounds) in the soil. Therefore, efforts are made to remove such substances in order to prevent the migration of harmful compounds to the groundwater, which is the cause of environmental pollution.

The fairly widespread use of explosives and their modified products has led to an in-crease in environmental pollution (especially soil, surface water and groundwater) to the extent of threatening human health, which, over the years, has become quite serious and it requires attention from the scientists [1]. One of the most commonly used explosives in the military is TNT (TNT) [2,3]. Nitroaromatic explosives are also widely used in the civil industry and mining [4,5]. The residues of TNT in the environment are toxic, can damage organs (i.e., liver, eyes, nervous system, circulatory system) and contribute to diseases such as anemia and cataract development [6]. There is information in the literature about the adverse effects of TNT not only on people but also on different ecosystems, e.g., microorganisms, plants, algae invertebrates, some vertebrates [7]. It is also toxic to aquatic life with long-lasting effects. Studies done on animals like mice, dogs and frogs have shown that TNT and its conversion products are cytotoxic, teratogenic and possibly mutagenic [8,9]. Due to the undoubtedly negative impact of explosives and their modified products on humans and animals, a new method of getting rid of these pollutants is still being sought from the environment [10,11,12,13].

Contemporary anti-personnel and anti-armor mines are mostly made of mineral fiber-reinforced TNT or have lightweight plastic casings such as PVC. Such a design makes it impossible to detect them with the most commonly used devices, i.e., metal detectors. In order to be able to detect such mines under the soil layer, scientists are working on new technologies to increase the sensitivity of the detection of volatile compounds of explosive substances, such as, for example, TNT. The most popular detection is the detection of vapors of explosives by dogs. They detect explosives with a sensitivity of 10^−16^ g/cm^3^ for TNT and 2,4-dinitrotoluene (DNT). Unfortunately, the work of dogs is very much influenced by environmental and weather conditions, so there is a need to train them in the conditions in which they will work [14]. Hence, the use of portable devices for detecting vapors of explosives is becoming more and more popular.

In order to choose the appropriate chemical method for mine detection, you first need to answer the question of what the mine smells like. The buried mine emits a mixture of various compounds into the environment through scratches and cracks in its housing, and also by diffusion from the plastic housing. Some of them can reach the earth’s surface and be the basis for the detection of mines. Their characteristics depend on factors such as temperature, pressure, air humidity and the type of substrate. So far, research has been carried out to check the composition of the vapors coming out of the mine. Analysis made by the U.S. Army Corps showed the presence of 2,4-DNT, 2-amino-4,6-DNT (2ADNT) and 4-amino-4,6-DNT (4ADNT) [15]. Similarly, researchers from the Swedish Defense Research Agency (FOI) dealt with the analysis of vapors from the surface above buried mines. The analysis of minefield samples from Bosnia and Herzegovina and Cambodia showed the lack of TNT pairs, while DNT and DNB pairs were present, additionally amino-DNT pairs were present in the samples from Cambodia [16].

To sum up, the studies of soil samples in which various types of anti-personnel mines were placed so far showed the highest concentration of 2,4-dinitrotoluene, 1,3-dinitrobenzene, and then 2,4,6-trinitrotoluene (TNT), as well as two compounds formed as a result of the environmental impact on TNT, i.e., 2-ADNT and 4-ADNT [17,18,19]. The greater presence of 2,4-DNT than TNT results from the differences in their vapor pressure—DNT—1.4 ng/l, TNT—70 ng/l, and it is also a result of the half-life in the soil (DNT—26 days at the temperature of 22 °C, TNT—24 h under similar conditions) [20,21].

It is also extremely important to have knowledge of how traces of explosives remain on the surface [22]. Durability and volatility of nitroglycerin (NG) means that it can be lost from the surface quite quickly and there is a risk that no traces will be found even after a short time. Similarly, most remains of a firearm are lost from hand after about 4 h [23]. On the other hand, e.g., hexogen (RDX) will stay on the surface for a very long time. This means that the time factor in interpreting explosive traces is very important.

**Figure 1:**
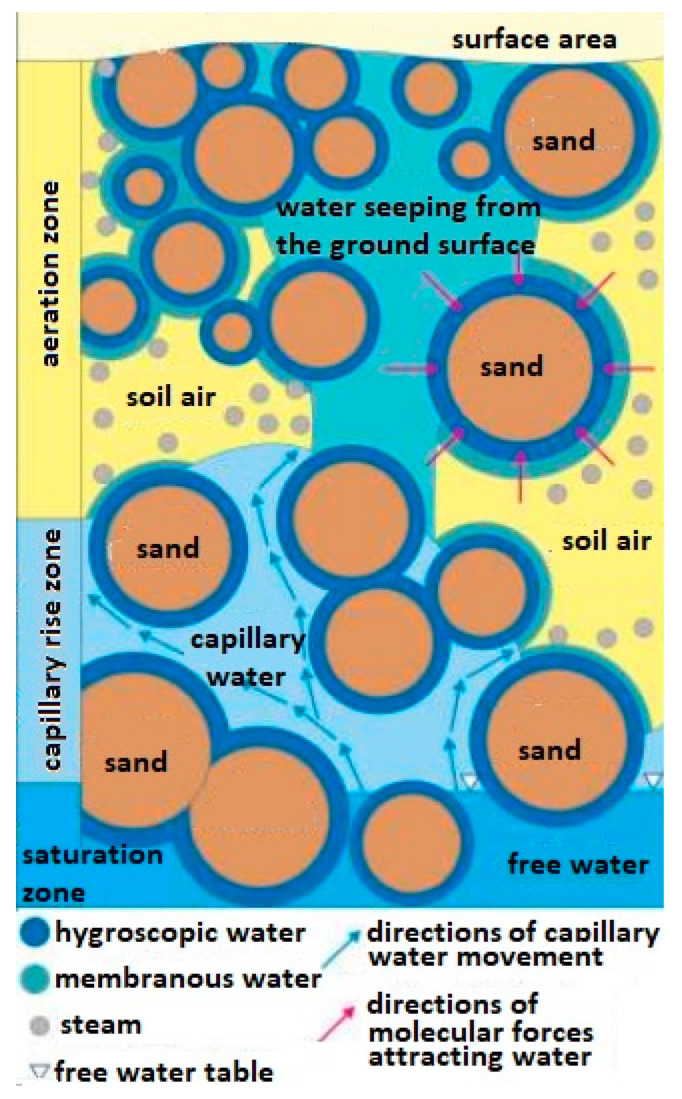
Diagram of types of water occurring in the ground. Source—educational materials Sławomir Dmowski.

The method of transporting water in the soil in which mines are hidden and whose chemical compounds of the explosive material can be transported this way is also important. It is assumed that in the soil substrate consisting of grains, e.g., sand, there is water in the form of steam [24,25] and in a bound form (hygroscopic and epithelial and capillary) [26,27] as well as free (ground and rainfall) [28] (Figure 1).

The membranous water (weakly bound water) is divided into fixed membrane water and loose membrane water. It is less bound to the surface of the particle, and it moves from one particle to another regardless of the force of gravity until the water thickness is equal on both particles. Membrane water is a layer with a thickness of 20 to 200 water particles. The thickness of the water film around the quartz grains with a diameter of 0.1 ÷ 0.05 mm is approx. 34 × 10^−6^ mm, and for particles 0.01 ÷ 0.005 mm—approx. 5 × 10^−5^ mm and does not transfer hydrostatic pressure.

In the available literature, it is difficult to find information on the exact location from which samples should be taken for searching for explosive residues at the site of a bomb attack. Currently, experimental work is being carried out in the world on the development, adaptation and implementation of portable detectors of explosive vapors for effective detection of non-metal mines. One of the parameters to consider is the substrate moisture content, which has a strong influence on the detection level of explosives vapors. The ranges of vapor concentration over plastic and metal mines in dry and wet soil are presented by J. MacDonald et al. [14]. From their work it can be concluded that the detector of explosive vapor should have a sensitivity of 10^−15^ g/cm^3^ for dry soil and 10^−12^ g/cm^3^ for wet soil in order to be able to detect non-metal mines.

T. F. Jenkins et al. in their work dealt with the influence of external factors on the concentration of vapors coming from inside mines to the outside [29]. The samples were prepared in three types of substrate—sand, loam and clay. Their measurements showed that both the temperature and humidity as well as the type of substrate have a significant impact on the concentration of vapors of explosives coming out of mines. They observed that in the sandy substrate, TNT, DNT and DNB pairs can be detected, and in the case of clay, no explosive vapors at all were detected. Another group studied the influence of weather conditions on mine detection by dogs [30]. They found that the lower the temperature, the less vapors are transported to the surface, and that the transport of TNT and DNT is greater in sand than in clay. In addition, they also drew attention to the fact that the humidity of the ground may affect the concentration of explosive vapors. They observed that in dry soils there is a strong adsorption and the vapor concentration is lowered. On the other hand, too much rainfall may cause the total washing away of the explosive material from the soil surface, thus reducing the vapor concentration.

As part of this article, the authors decided to investigate the influence of substrate moisture on the detection of TNT vapors using the popular MO-2M explosive vapor detector, the detection mechanism of which is based on the FAIMS method. This detector uses the principle of the nonlinear dependence of the mobility of ions on the electric field strength. The devices operate on the basis of an internal standard in the form of, e.g., 2,4,6-trinitrotoluene, which enables further analysis and identification. When explosive pairs are detected, a signal appears, and a spectrum (ionogram) is obtained, showing the dependence of the ionic current on the compensating voltage.

For this purpose, a sandy substrate was selected, as it is the most common substrate in the present theater of hostilities, mainly in countries with a dry and hot climate. Besides, sand is the best-characterized material for this type of research. The authors consider soaking the sand with water that ensures the presence of water capable of moving towards the surface in the form of water vapor and water bound to the substrate grains in the form of membrane water (weakly bound).

Tests were carried out to show the influence of humidity on TNT transport from under the sand layer to its surface. In addition, it was determined after what time the detector alarm, indicating the presence of TNT vapors, appears and how it affects the thickness of the sand layer and the temperature of the substrate. The obtained results were used to assess the influence of humidity on the concentration of vapors on the surface of the substrate and the possibility of estimating the optimal time for TNT detection in sand.

## 2. Experimental

### 2.1. Materials

The research used TNT, which is the most commonly used military explosive. It is very often used in many types of mines. Its universality is due to its high stability, low sensitivity to mechanical stimuli and a relatively safe production method. TNT as a material with an average vapor concentration (9.5 ppb at 25 °C) is relatively easy to detect using explosive vapor detectors [31]. The mentioned vapor pressure of explosives depends on the ambient temperature (it increases with increasing temperature). For TNT, it doubles with each additional 5 °C increase in temperature [32].

#### 2.1.1. Preparation of Sand for Testing

The research was carried out on sandy ground. Construction sand with a grain size of 0.1 to 0.3 mm and a bulk density of 1.6 g/cm^3^ was selected for the tests. This sand contained residues of organic compounds as well as their decomposition products, therefore the MO-2M detector gave false positive alarms. Therefore, it was necessary to remove organic impurities so that when testing the samples with sand, there was no reaction of the device. For this purpose, the sand was calcined in a metal pot at 350–400 °C for 1 h. After cooling, the sand was checked with the MO-2M detector and it showed no residual organic compounds, wrongly identified as MW. The sand prepared in this way was stored under a cover, in a clean room, with no access to explosives.

#### 2.1.2. Preparation of Sand Samples

The preparation of sand samples was carried out in three stages.

#### 2.1.3. Stage 1—Adaptation of the Workplace

Thorough checking of the workplace and the tools used with the MO-2M detector. If the detector revealed the presence of explosives, these elements were washed and the operation was repeated until no alarm was obtained on the detector. Measurements were carried out on previously unfolded paper.

#### 2.1.4. Stage 2—Preparation of Containers

Jars with a capacity of 770 mL were used as containers for sand, which were washed first with detergent and then with acetone, dried and checked with the MO-2M detector for the presence of explosives or false positives signals. These actions were repeated until there was no alarm on the detector. A scale was marked on the clean jars, which indicated to what level the sand should be poured.

#### 2.1.5. Stage 3—Placing TNT in Containers

Sand was poured into a clean jar to the marked level and then very carefully (without staining its walls) a TNT pellet with a weight of 5 g and an area of approx. 8 cm^2^ was placed on the surface. Then, sand was refilled to the marked level. The samples prepared in this way were left covered with crystallizers in the fume cupboard until the tests began.

**Figure 2:**
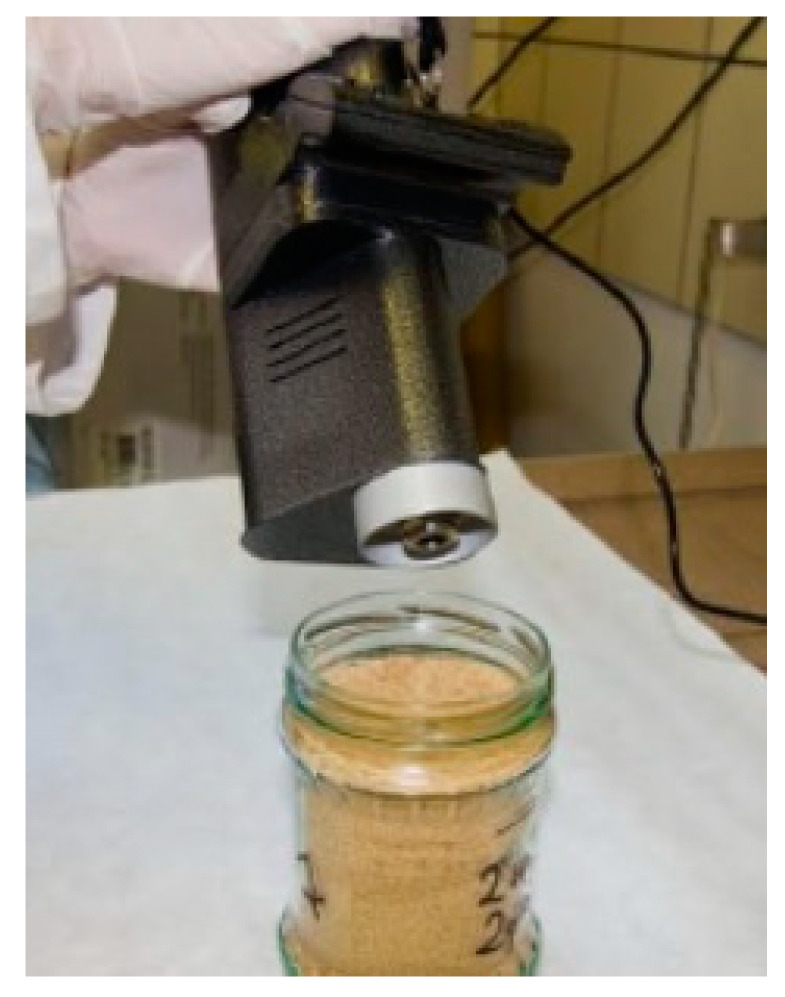
Photo of the sand jar showing the location of the MO-2M detector inlet nozzle.

Before starting the tests, the conditions in the room were measured, i.e., temperature, pressure and humidity. After the MO-2M detector was turned on and its operation stabilized, samples in the form of TNT embedded in sand were flooded with a specific amount of water. The measurement was made by bringing the detector inlet nozzle closer to the distance of 0.5 cm from the sand surface in each tested sample for 10–15 s (Figure 2). In turn, the arithmetic mean of the three measurements was taken as the maximum average level.

### 2.2. Methods

#### 2.2.1. Detection of Explosives Vapors Using the FAIMS Method—MO-2M Detector

Ion mobility spectrometry (IMS) is the most frequently used technique for detecting explosives. To detect TNT pairs, the MO-2M detector was used, which uses the method of non-linear dependence of ion mobility on the electric field strength (FAIMS) [33,34,35,36]. This method measures changes in the mobility of individual ions [37]. In the first stage, the explosive vapors are ionized by an ionization source and then separated in the drift region. For specific ions, the drift trajectory is different due to the different dependence on the electric field strength [38]. This detector is characterized by a very high sensitivity, allowing us to detect the presence of explosive substance vapors even at a concentration below 10^−13^ g/cm^3^ [39]. After starting the device, the parameters are automatically adjusted to the environmental conditions (auto-calibration). During the measurement, the air above the surface of the test object is drawn through the inlet nozzle at a speed of 1 cm^3^/s.

#### 2.2.2. Sand Extract Test

The sand (in the amount of about 10 g) collected from the sample surface was washed three times with methanol and the extract thus obtained was analyzed. GC Agilent Technologies 7890A gas chromatograph with MS 5975C mass detector was used to perform the analyses.

Operating conditions in the gas chromatograph for the analysis:

Column type HP-5MS 30 m × 250 µm × 0.25 µm was used. The flow rate of carrier gas (helium) was set on 1 ml/min. Sample injections volume was 1 µL. Split ratio was 1:50. A single quadrupole was used.

The analyses carried out using the temperature program:

50 °C for 1 min

Increase of 2.5 °C/min to 70 °C

Increase of 20 °C/min to 280 °C

280 °C for 20 min

#### 2.2.3. Investigation of the Influence of Substrate Humidity on the Detection of TNT Vapors

During the measurements, the cleanliness of the measuring station and the used tools were taken care of. Clean protective gloves were worn before each measurement. Jars with TNT at a depth of 0.5 cm from the sand surface were used for the tests. The samples were prepared one week before starting the measurements. The sand was moistened with 5%, 10% and 15% by mass of water, respectively. The method of carrying out the measurement series was as follows:

1. Place the jar on the test stand;
2. Bring the detector inlet nozzle closer to 0.5 cm from the sand surface for 10–15 s; and
3. Move the detector away from the jar for 10–15 s at a distance not less than 1 m.

Steps 2 and 3 were repeated three times, and then the jar was returned to the storage in an airy place. Measurements were taken until the water was completely lost from the substrate.

#### 2.2.4. Study of the Influence of Temperature on the TNT Detection from under the Sand Layer

The cleanliness procedure during sample preparation was the same as for the study of the influence of the amount of water on the detection of TNT vapor. Jars with TNT at a depth of 0.5 and 2.5 cm were used for the tests. The sand was moistened with 10% by mass of water. The tests were carried out for samples prepared one week before the substrate was moistened. The method of carrying out the measurement series was as follows:

1. The jars were heated to 50 °C using halogen lamps;
2. The jars were placed on the measuring stand;
3. Bring the detector inlet nozzle closer to the sand surface, 0.5 cm distance, for 10–15 s; and
4. Move the detector away from the jar for 10–15 s at a distance of not less than 1 m.

Steps 2 and 3 were repeated three times and then the jar was set aside to reheat. Measurements were taken until the water was completely lost from the substrate.

#### 2.2.5. Analysis of Measurement Errors

The results could be influenced by the conditions in the room where the fume cupboard was located, under which the experiments were carried out. Therefore, every effort has been made to ensure that these conditions remain largely unchanged. In order to determine the reliability and repeatability of the obtained results, we used the so-called mean square error, i.e., the standard deviation of a single measurement, thus determining its uncertainty.

## 3. Results and Discussion

### 3.1. Investigation of the Influence of Substrate Humidity on the Detection of TNT Vapors

The test results were prepared in the form of graphs illustrating the change in the average level of TNT signal with time depending on the amount of water contained in the substrate for the TNT ingot placed 0.5 cm below the sand surface.

**Figure 3:**
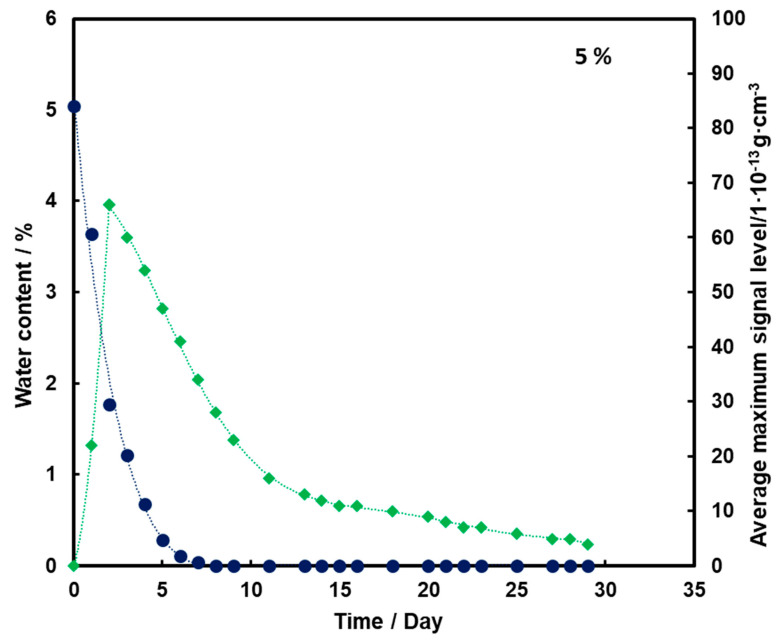
Dependence of the mean maximum TNT signal (♦) on the amount of water (●) in time for a substrate with a moisture content of 5%.

By analyzing Figure 3, it can be observed that as the substrate moisture decreases, there is an initial increase (up to day 2) and then a logarithmic decrease in the mean TNT signal. In addition, the TNT signal is observed even after the water has completely disappeared from the medium.

**Figure 4:**
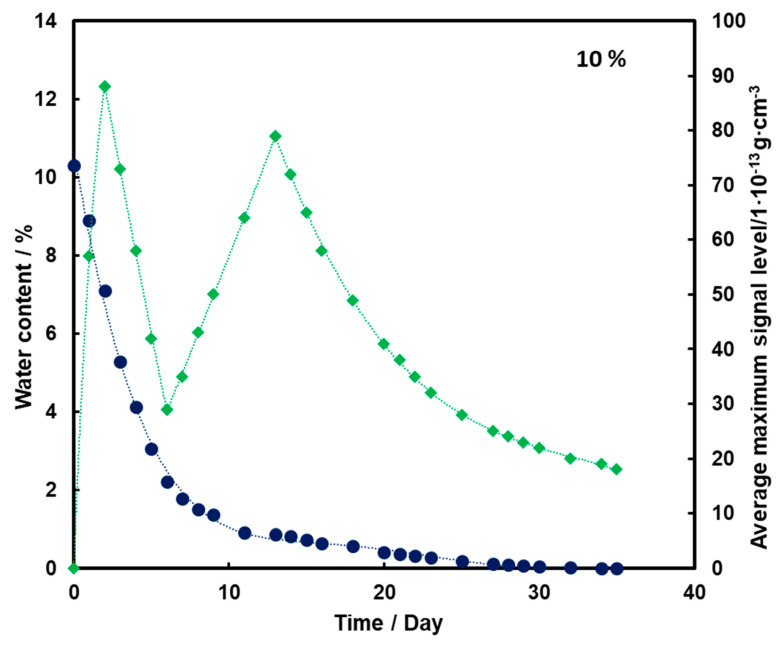
Dependence of the mean maximum TNT signal (♦) on the amount of water (●) in time for a substrate with a humidity of 10%.

**Figure 5:**
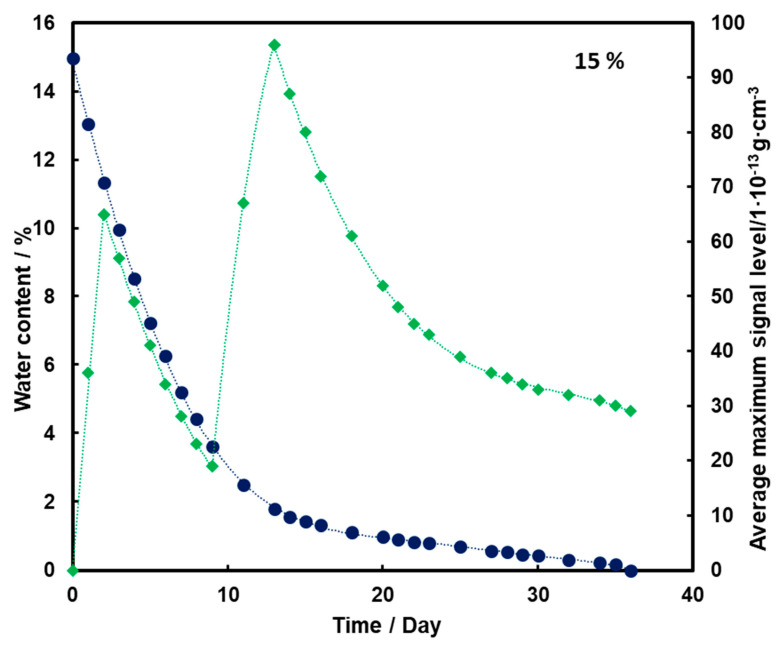
Dependence of the mean maximum TNT signal (♦) on the amount of water (●) in time for a substrate with a moisture content of 15%.

For the substrate with a moisture content of 10% and 15% (Figure 4 and Figure 5, respectively), an initial increase in the mean TNT signal is also observed with the loss of water from the substrate (also up to the second day). However, a decrease and a further increase in mean TNT signal are observed. In both cases, only after the 13th day of measurements, a logarithmic decrease in the mean TNT signal is observed. However, this decrease is less than that for a sample with a moisture content of 5%.

For all three subsoil humidity values, we observe a similar dependence of the maximum mean TNT signal in time—the TNT signal is recorded throughout the duration of the measurements. On the other hand, it should be noted that for the sample with 5% water content, the TNT signal reaches one maximum, while for the samples with 10% and 15% water content, two maximums are observed. This is because, depending on the amount of water in the substrate, different transport of TNT is observed. When there is little moisture in the substrate (5%), only transport of TNT with water vapor is observed. According to the rheology of water, there is also transport of TNT dissolved in the membranous water in the squeak, but in this case this effect is not observed, because there is not enough water in the sample to allow it to move in this way. In samples with more water (10% and 15%), transport by evaporation of TNT with water vapor is also observed (first maximum), but there is also an effect related to transport of TNT through loosely bound water to the surface of the sand layer (second maximum). Initially, TNT travels with water vapor, and at the same time, TNT partially dissolved in water is transported in an aqueous solution, moving from one grain to another with the water films to the surface. It concentrates there, because its content in water is much greater than the possibility of evaporation, which causes it to crystallize and then only slowly evaporate from the surface. The second maximum occurs when the evaporation of TNT with water vapor decreases (the substrate dries up) and the amount of concentrated TNT on the surface is large. The weathering (logarithmic loss) of solid TNT concentrated on the sand is then observed.

Understanding and knowledge of the TNT transport and evaporation processes occurring in the sandy substrate has become useful in determining when is the most optimal time to look for TNT in the substrate. From the analysis of the obtained results, it can be concluded that the most advantageous search for TNT vapors would be as many days after the rain for the sand moisture content to be at least 10%.

Trace amounts of TNT were found on the wet sand surface. Due to the fact that no significant differences were observed in the course of the dependence of the average maximum signal on the amount of water in the substrate in time for 10% and 15%, it was decided that in further measurements the sand would be moistened with 10% by mass of water.

### 3.2. Study of the Influence of Temperature on the TNT Detection from under the Sand Layer

In order to check the influence of temperature on the detection of TNT vapors, the experiment was carried out at two temperatures—ambient temperature of approx. 25 °C and at an elevated temperature of about 50 °C. The temperature range assumed in the experiment is related to the real conditions that most often occur in reality in the environment (higher temperatures than 50 °C rarely occur in natural conditions; at temperatures lower than 25 °C, the vapor pressure of explosives is so low that the possibility of their detection is reduced). The obtained results are presented in the form of graphs showing the change in the mean level of the TNT signal located at 0.5, 2.5 and 5 cm, respectively, below the sand surface, taking into account the change in the amount of water contained in the substrate.

#### 3.2.1. Ambient Temperature

The results obtained for the TNT pellet placed 0.5 cm below the sand surface are presented and discussed earlier in Figure 4.

**Figure 6:**
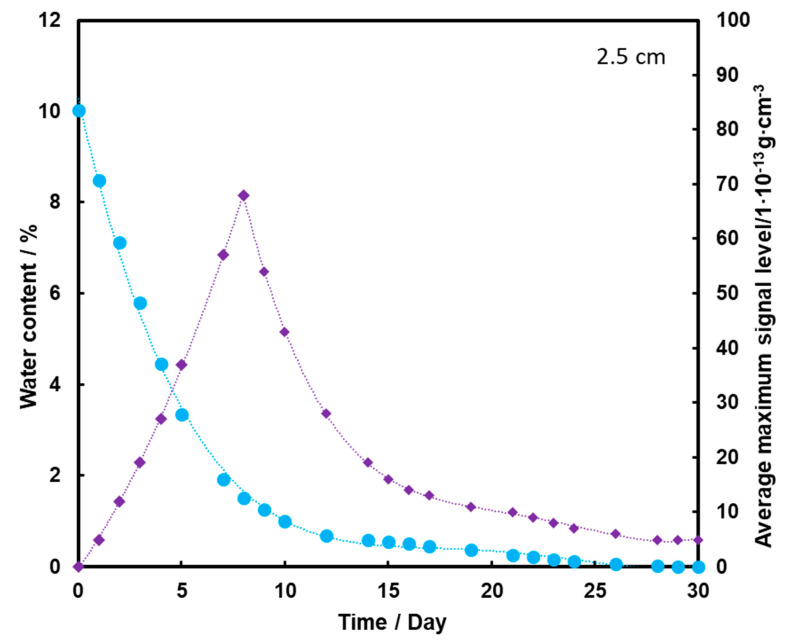
Dependence of the mean maximum TNT signal (♦) on the amount of water (●) in the substrate for the TNT sample at a depth of 2.5 cm, at a temperature of 25 °C.

In Figure 6, an initial increase in the TNT signal is observed to a maximum occurring on the 8th day of measurement. Then, its logarithmic decrease is observed, but even after 30 days the signal is registered by the detector. Transport of TNT to the sand surface is in this case associated with transport with water vapor. Even when there is no more water in the substrate, small amounts of TNT still remain on the surface of the sand.

**Figure 7:**
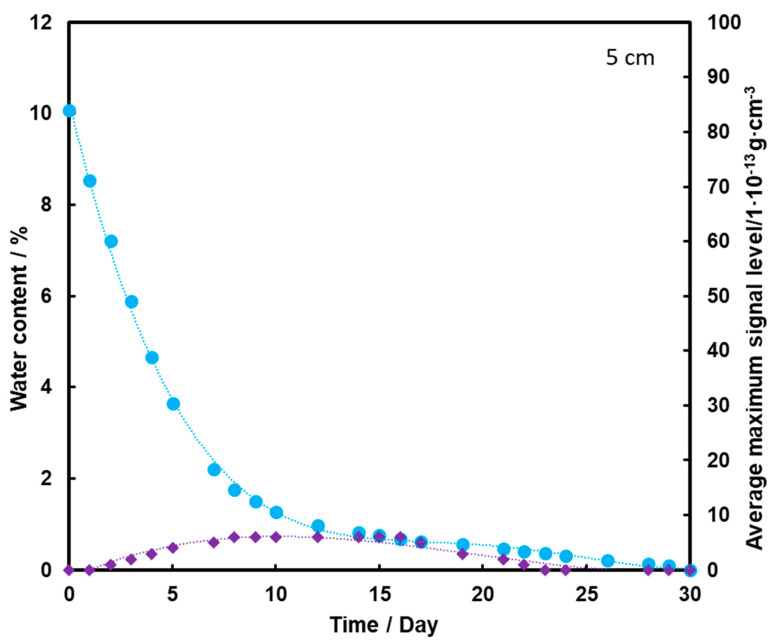
Dependence of the mean maximum TNT signal (♦) on the amount of water (●) in the substrate for the TNT sample at a depth of 5 cm, at a temperature of 25 °C.

Figure 7 shows that the TNT signal appears on the second day of measurement, but it is at a fairly low level. The maximum is reached on the 8th day of measurements, and then it drops to zero after the 20th day of measurements. Comparing the presented graphs, it can be seen that the deeper the TNT pill is located under the sand layer, the weaker the TNT signal we have. Thus, a thicker layer of sand contributes to the retention of a large part of the transported TNT on the sand grains and TNT, moving with steam, and does not reach the surface of the substrate with water. The transport effect of TNT with water vapor is hardly ever observed in this case.

#### 3.2.2. Increased Temperature

The results of the tests obtained for TNT placed 0.5 and 2.5 cm below the surface of the sand were prepared in the form of graphs showing the dependence of the change in the average TNT signal level with time, taking into account the amount of water in the substrate. For samples with TNT placed 5 cm below the surface of the sand, no signal was obtained on the MO-2M detector for the entire duration of the experiment (until the sand was completely lost).

**Figure 8:**
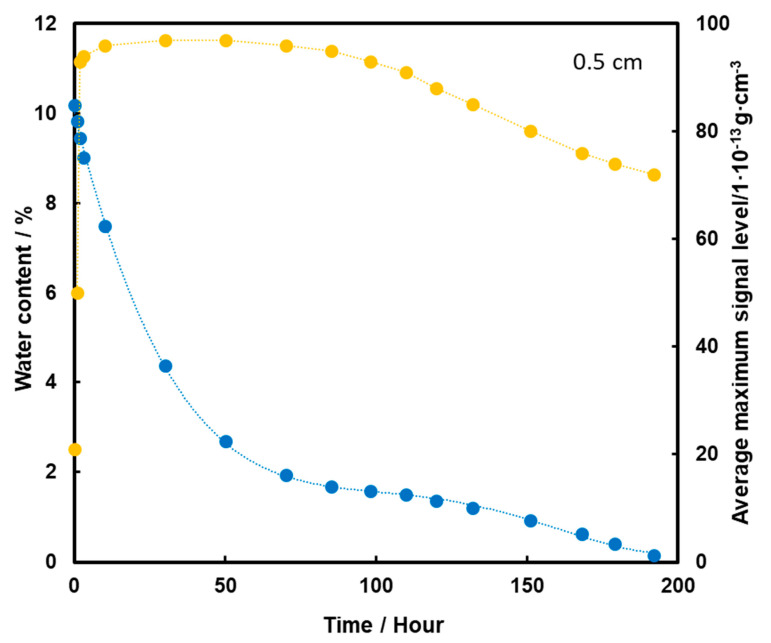
Dependence of the mean maximum TNT signal (●) on the amount of water (●) in the substrate for the TNT sample at a depth of 0.5 cm, at a temperature of 50 °C.

TNT placed at a depth of 0.5 cm is detected from the start of the measurements (Figure 8). After 30 h of heating, the TNT signal has reached its maximum, which is extended to almost 100 h, i.e., about 4 days. As a result of further heating, the level of the TNT signal decreases slightly, but is still quite high despite the rapid loss of water from the substrate. The high level of signal after evaporation of water entirely from the sample may indicate that TNT transport took place mainly by the flow of the aqueous solution with films on the squeak grains, which had a higher concentration of TNT due to its better solubility at higher temperatures. This caused more TNT crystals to precipitate on the surface of the screeching.

**Figure 9:**
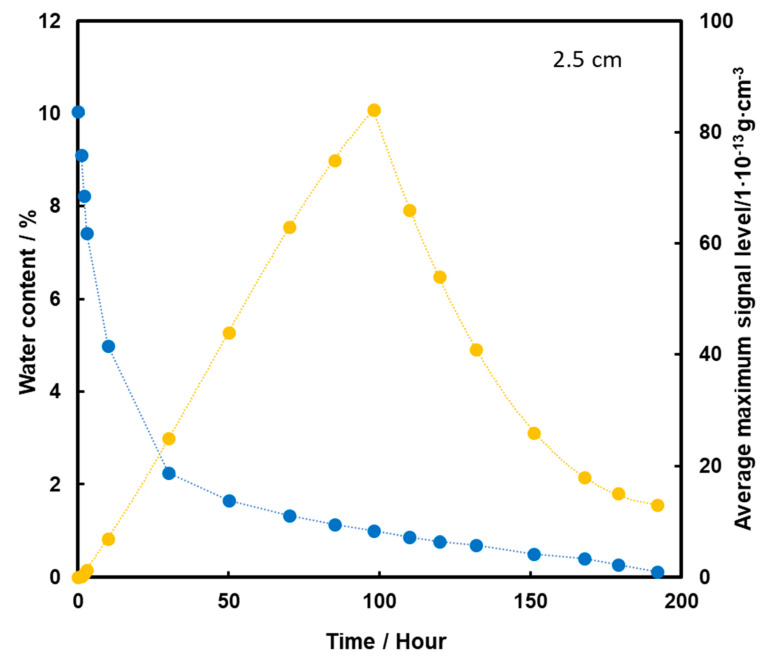
Dependence of the mean maximum TNT signal (●) on the amount of water (●) in the substrate for the TNT sample at a depth of 2.5 cm, at a temperature of 50 °C.

When TNT is placed 2.5 cm below the surface of the sand, an initial increase in the TNT vapor signal is observed with the loss of water from the substrate (Figure 9). The maximum is reached in 98 h, which is 4 days of heating, and then a decrease in TNT signal is observed. Quite a low level of TNT signal at the time of total loss of water from the sample may indicate that in this case, when the pellet is placed lower, the dominant mechanism of TNT transport to the surface is transport with water vapor.

## 4. Conclusions

The sandy substrate greatly suppresses the possibility of trace amounts of TNT escaping to the surface and for dry sand a layer of 0.5 cm effectively blocks the possibility of TNT detection with explosive vapor detectors based on the FAIMS technique, which detects TNT in the air at the level of 10^−13^ g.

On the basis of the obtained results, it should be concluded that the presence of water in the sandy substrate has a large impact on the possibility of detecting the TNT charge under its surface with the use of a detector of explosive vapor based on the FAIMS technique.

The results showed that trace amounts of TNT from the mine can reach the surface through the sand layer in two ways—gas-phase transport with water vapor and the movement of epithelial water on sand grains to grains with a thinner epithelial layer so that they balance, that is mainly towards the surface.

The moisture level of the sand and the depth of the TNT charge of the mine beneath the sandy surface determine the dominant mechanism of TNT transport to the surface, and, thus, the time after which we can detect trace amounts of TNT on the surface of the substrate. For higher sand moisture levels above 10% of water and the shallow location of the TNT charge of the mine (about one centimeter below the surface of the substrate), the time after which the maximum emission of TNT took place from the sand surface was 2 days. When the mine was located at a depth of several centimeters below the ground, the time to obtain the maximum emission level was up to several days and was related to the mechanism of transport of TNT traces by means of film-like water.

Increasing the temperature on a wet sandy substrate significantly accelerates the detection and extends the time of TNT emission from under the sand layer, which increases the level of TNT detectability.

Knowledge of how TNT is transported from the ground to the surface is very useful in estimating how long it would take to look for explosive vapors from TNT-charged mines to be able to effectively remove them from the environment.

## Data Availability

Data available on request due to restrictions eg privacy or ethical. The data presented in this study are available on request from the corresponding author.

## Sample Availability

Samples of the compounds are not available from the authors.

## References

[1] Fahrenfeld N., Zoeckler J., Widdowson A., Pruden A. (2013). Effect ofbi-ostimulants on 2,4,6-trinitrotoluene (TNT) degradation andbacterial community composition in contaminated aquifersediment enrichments. Biodegradation.

[2] Chien C.C., Kao C.M., Chen D.Y., Chen S.C., Chen C.C. (2014). Bio-transformation of trinitrotoluene (TNT) by Pseudomonas spp.iso-lated from a TNT-contaminated environment. Environ. Toxicol. Chem..

[3] Cho C., Bae S., Lee W. (2012). Enhanced Degradation of TNT and RDXby Bio-reduced Iron Bearing Soil Minerals. Adv. Environ. Res..

[4] Kulkarni M., Chaudhari A. (2007). Microbial remediation of nitroaromatic compounds: An overview. J. Environ. Manag..

[5] Mercimek H.A., Dincer S., Guzeldag G., Ozsavli A., Matyar F. (2013). Aerobic biodegradation of 2,4,6-trinitrotoluene (TNT) by Ba-cillus cereus isolated from contaminated soil. Microb. Ecol..

[6] Zhang H., Feng L., Liu B., Tong C., Lü C. (2014). Conjugation of PPV functionalized mesoporous silica nanoparticles with graphene oxide for facile and sensitive fluorescence detection of TNT in water through FRET. Dyes Pigm..

[7] Kuperman R.G., Checkai R.T., Simini M., Phillips C.T., Kolakowski J.E., Lanno R. (2013). Soil properties affect the toxicities of 2,4,6-trinitrotolu-ene (TNT) and hexahydro-1,3,5-trinitro-1,3,5-tria-zine (RDX) to the en-chytraeid worm Enchytraeus crypticus, Environ. Toxicol. Chem..

[8] Maeda T., Kadokami K., Ogawa H.I. (2006). Characterization of 2,4,6-Trinitrotoluene (TNT)-Metabolizing Bacteria Isolated from TNT-Polluted Soils in the Yamada Green Zone, Kitakyushu. Japan. J. Environ. Biotechnol..

[9] Mercimek H.A., Dincer S., Guzeldag G., Ozsavli A., Matyar F., Arkut A., Kayis F., Ozdenefe M.S. (2015). Degradation of 2,4,6-trini-trotoluene by P. aeruginosa and characterization of some me-tabolites. Braz. J. Microbiol..

[10] Bui D.N., Minh T.T. (2021). Investigation of TNT red wastewater treatment technology using the combination of advanced oxidation processes. Sci. Total Environ..

[11] Li J., Zhou Q., Li M., Liu Y., Song Q. (2021). Monodisperse amino-modified nanosized zero-valent iron for selective and recyclable removal of TNT: Synthesis, characterization, and removal mechanism. J. Environ. Sci..

[12] Nhi B.D. (2021). Research on Regenerating Activated Carbon in 2,4,6-Trinitrotoluene (TNT) Explosives Manufacturing Industry by Microwave Radiation and Ionized Nitrogen. Propellants Explos. Pyrotech..

[13] Tian R., Ji P., Wang L., Zhang H., Sun J. (2021). TNT sensor based on accumulation layer and effective distance of FRET mechanism with ultra-high sensitivity. Microchem. J..

[14] MacDonald J., Lockwood J.R., McFee J., Altshuler T., Broach T., Carin L., Harmon R., Rappaport C., Scott W., Weave R. (2003). Alternatives for Landmine Detection.

[15] Leggett D.C., Cragin J.H., Jenkins T.F., Ranney T. (2000). Release of Explosive-Related Vapors from Land Mines.

[16] Kjellström A.H., Sarholm L.M., Dubey A.C., Harvey J.F., Broach J., Dugan R.E. (2000). Analysis of TNT and related compounds in vapor and solid phase in different types of soil. Detection and Remediation Technologies for Mines and Minelike Targets V.

[17] George V., Jenkins T.F., Leggett D.C., Cragin J.H., Phelan J., Oxley J., Pennington J. (1999). Progress on Determining the Vapor Signature of a Buried Landmine. Detection and Remediation Technologies for Mines and Minelike Targets IV.

[18] Jenkins T.F., Walsh M.E., Miyares P.H., Kopczynski J.A., Ranney T.A., George V., Pennington J., Berry T.E. (2000). Analysis of Explosives-Related Chemical Signatures in Soil Samples Collected near Buried Land Mines.

[19] Leggett D.C., Jenkins T.F., Murrmann R.P. (1977). Composition of Vapors Evolved from Military TNT as Influenced by Temperature Solid Composition, Age, and Source.

[20] Schubert H., Kuznetsov A. (2002). Detection of Explosives and Landmines, Methods and Field Experience.

[21] Miyares P.H., Jenkins T.F. (2000). Estimating the Half-Lives of Key Components of the Chemical Vapor Signature of Land Mines.

[22] Caddy B. (2001). Trace evidence—Small samples, big problems. Probl. Forensic Sci..

[23] Jalant T., Henchoz P., Gallusser A., Bonfanti M.S. (1999). The persistence of gunshot residue on shooters’ hands. Sci. Justice.

[24] Janssen H. (2011). Thermal diffusion of water vapour in porous materials: Fact or fiction?. Int. J. Heat Mass Transf..

[25] Philip J.R., de Vries D.A. (1957). Moisture movement in porous materials under temperature gradient. Trans. Am. Geophys. Union.

[26] Giedrojć B. (1974). Metodyka badań kapilarnego potencjału wodnego zróżnicowania porowatości gleb częściowo zmodyfikowanym kapilarymetrem Sekery. Rocz. Glebozn..

[27] Wysocka M., Szypcio Z., Tymosiak D. (2013). Capillary rise speed in non-cohesive soils. Civil Environ. Eng..

[28] Coduto D.P., Yeung M.R., Kitch W.A. (2011). Geotechnical Engineering: Principles and Practices.

[29] Jenkins T.F., Legget D.C., Ranney T.A. (1999). Vapor Signature from Military Explosives—Part I: Vapor Transported from Buried Military-Grade TNT.

[30] Phelan J.M., Webb S.W., Rodacy P.J., Barnett J.L. (2001). Environmental Impact to the Chemical Signature Emanating from Buried Ordnance—Final Report.

[31] Rhykerd C.L., Hannum D.W., Murray D.W., Parmeter J.E. (1999). Guide for the Selection of Commercial Explosives Detection Systems for Law Enforcement Applications.

[32] Mostak P., Krausa M., Alekseyvitch Reznev A. (2004). Vapour and trace detection of explosives. Vapour and Trace Detection of Explosives for Anti-Terrorism Purposes.

[33] Keller T., Keller A., Tutsch-Bauer E., Monticelli F. (2006). Application of ion mobility spectrometry in cases of forensic interest. Forensic Sci. Int..

[34] Kolakowski B.M., Mester Z. (2007). Review of applications of high-field asymmetric waveform ion mobility spectrometry (FAIMS) and differential mobility spectrometry (DMS). Analyst.

[35] Pawłowski W., Zalewska A., Matyjasek Ł., Karpińska M. (2017). The air humidity effect on the detection of TNT, PETN and NG by the FAIMS technique. Sens. Actuat. B Chem..

[36] Zalewska A., Pawłowski W., Tomaszewski W. (2013). Limits of detection of explosives as determined with IMS and field asymmetric IMS vapour detectors. Forensic Sci. Int..

[37] Pettersson A., Wallin S., Brandner B., Eldsäter C., Holmgren E. (2006). Explosives Detection—A Technology Inventory.

[38] Shvartsburg A.A. (2009). Differential Ion Mobility Spectrometry, Nonlinear Ion Transport and Fundamentals of FAIMS.

[39] SIEBEL, Internal Materials by SIBEL Ltd. (2011). MO-2M—Portable Detector of Explosive Vapors. Physical Foundations of NZPI Technology. http://www.sibel.info/en/explosives-detectors/mo-2m.html.

